# Thermodynamics and Spectroscopy of Halogen- and Hydrogen-Bonded Complexes of Haloforms with Aromatic and Aliphatic Amines

**DOI:** 10.3390/molecules27186124

**Published:** 2022-09-19

**Authors:** Emmanuel Adeniyi, Olivia Grounds, Zachary Stephens, Matthias Zeller, Sergiy V. Rosokha

**Affiliations:** 1 Department of Chemistry, Ball State University, Muncie, IN 47306, USA; 2 Department of Chemistry, Purdue University, West Lafayette, IN 47907, USA

**Keywords:** halogen bonding, hydrogen bonding, haloforms, Amines, NMR spectroscopy, UV–Vis spectroscopy, DFT calculations, X-ray crystallography

## Abstract

Similarities and differences of halogen and hydrogen bonding were explored via UV–Vis and ^1^H NMR measurements, X-ray crystallography and computational analysis of the associations of CHX_3_ (X=I, Br, Cl) with aromatic (tetramethyl-*p*-phenylenediamine) and aliphatic (4-diazabicyclo[2,2,2]octane) amines. When the polarization of haloforms was taken into account, the strengths of these complexes followed the same correlation with the electrostatic potentials on the surfaces of the interacting atoms. However, their spectral properties were quite distinct. While the halogen-bonded complexes showed new intense absorption bands in the UV–Vis spectra, the absorptions of their hydrogen-bonded analogues were close to the superposition of the absorption of reactants. Additionally, halogen bonding led to a shift in the NMR signal of haloform protons to lower ppm values compared with the individual haloforms, whereas hydrogen bonding of CHX_3_ with aliphatic amines resulted in a shift in the opposite direction. The effects of hydrogen bonding with aromatic amines on the NMR spectra of haloforms were ambivalent. Titration of all CHX_3_ with these nucleophiles produced consistent shifts in their protons’ signals to lower ppm values, whereas calculations of these pairs produced multiple hydrogen-bonded minima with similar structures and energies, but opposite directions of the NMR signals’ shifts. Experimental and computational data were used for the evaluation of formation constants of some halogen- and hydrogen-bonded complexes between haloforms and amines co-existing in solutions.

## 1. Introduction

While hydrogen bonding (HB) has been extensively studied for more than a century [1,2], similar interactions involving other atoms have captivated the attention of the chemical community only during the last two decades [3,4,5]. Halogen bonding (XB) is a prominent example of these newly recognized interactions. It has already become a powerful tool for molecular recognition, crystal engineering, catalysis and many other applications [6,7,8]. The ubiquity of molecules containing both hydrogen and halogen substituents and the similarity in the factors determining XB and HB strength implies that many of them might be involved in both types of interactions. In fact, many X-ray structural studies revealed a co-existence of XB and HB bonds, or a dominance of one of them in co-crystals of such molecules with various nucleophiles [9,10,11,12,13]. However, the experimental characterization of these competing or complementary intermolecular interactions in solutions represents a challenging task which requires an accurate knowledge of the distinctions between XB and HB complexes formed by the same molecule with the same nucleophile.

**Figure 1:**
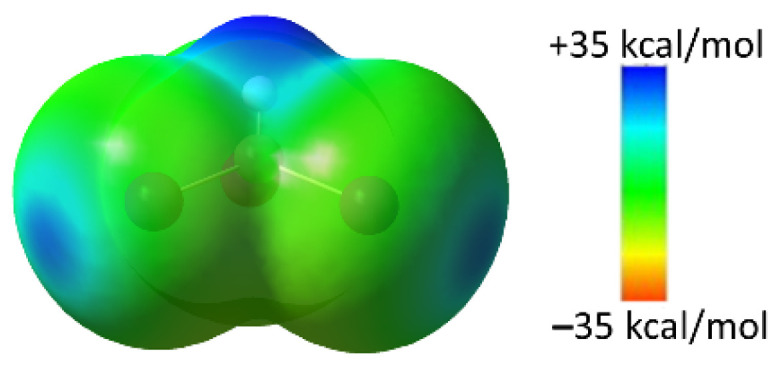
Surface electrostatic potential of iodoform (at 0.001 a.u. electron density) showing areas of positive potentials (σ-holes) along the extensions of C–H and C–I bonds.

The haloforms, CHX_3_ (X=I, Br or Cl) are characterized by the areas of positive potentials (σ-holes) along the extensions of the C–H and C–X bonds (Figure 1) [14]. As such,

These simple molecules can form both HB and XB complexes via the attraction of nucleophiles (Lewis base) B to their halogen or hydrogen substituents (Equations (1) and (2)):

(1) $${\mathrm{CHX}}_{3}+B\;\overset{K_{\mathrm{XB}}}{⇄}{\;[X}_{2}\mathrm{HC}\text{-}X\cdots B]$$

(2) $${\mathrm{CHX}}_{3}+B\;\overset{K_{\mathrm{HB}}}{⇄}{\;[X}_{3}C\text{-}H\cdots B]$$

where K_XB_ and K_HB_ are formation constants of the corresponding complex. Most frequently, such complexes were studied using NMR spectroscopy [15,16,17,18]. The earlier NMR studies of the complexes of halides with haloforms by Green and Martin showed that CHI_3_ forms predominantly XB complexes in which the signal of the proton is shifted to lower ppm values [17]. The HB associations (in which NMR signals of the haloforms’ protons are shifted to higher values) prevail in the solutions of halides with CHBr_3_ or CHCl_3_. Similar conclusions were obtained by Bertrán and Rodrígues based on NMR studies of the interaction of haloforms with aza-containing solvents [18]. In the recent study by Schulz et al., the relative strengths of the XB/HB interactions of haloimidazolium derivatives were measured experimentally, and the quantitative comparison of the interaction energies and free energies of different association modes were derived from quantum mechanical calculations and molecular dynamics simulations [19].

**Scheme 1:**
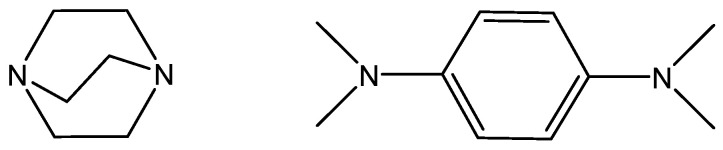
Structures of DABCO (**left**) and TMPD (**right**).

UV–Vis spectroscopy represents another method with a high potential for the differentiation of HB and XB interactions [14]. Our recent study showed that a combination of NMR and UV–Vis measurements together with computational analysis allows quantitative characterization of the concurrent XB and HB complexes between haloforms and (pseudo-)halide anions in solutions [14]. We have also demonstrated that the common anesthetic, halothane, acts as a XB and HB donor in solutions, which gives an atomic rationale for its eudismic ratio [20]. To establish generalities and limitations of the application of the NMR and UV–Vis spectroscopies for the identification and characterization of the co-existing XB and HB complexes, we turned in the current work to complex formation of haloforms with aromatic and aliphatic amines (which represent an important class of XB and HB acceptors). While the crystallographic literature contains a number of X-ray structures of either XB and HB complexes with these neutral nucleophiles [21,22,23,24,25], and these interactions were compared via computational analysis [26,27,28,29], the efforts of differentiating and characterizing the concurrent formation of the corresponding XB and HB complexes in solutions are lacking. As such, we carried out liquid-phase measurements and computational analysis of the interaction of haloforms with *N,N,N′,N′*-tetramethyl-p-phenylenediamine (TMPD) and 4-diazabicyclo[2,2,2]octane (DABCO) (Scheme 1).

The general character of the spectral features observed with these nucleophiles were verified via measurements of interactions of haloforms with other aliphatic and aromatic amines (trimethylamine and various *N,N*-dimethylanilines). Such a study facilitates the identification and quantitative characterization of the co-existing XB and HB complexes involving the same pair of reactants in chemical and biochemical systems, and clarification of the factors which determine the strengths and preferences of one or another mode of interaction.

## 2. Results and Discussion

### 2.1. UV–Vis Study of Interaction of CHX_3_ with Amines

**Figure 2:**
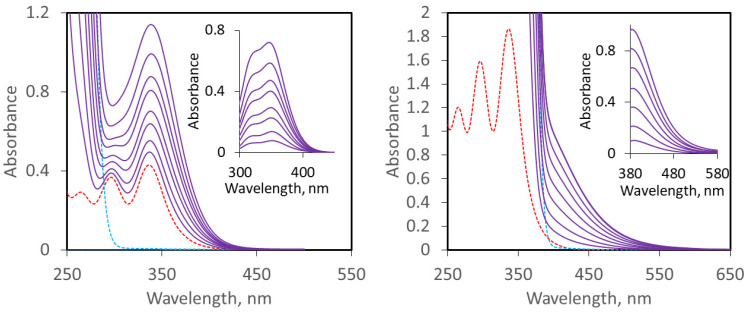
Spectra of solutions with a constant concentration of CHI_3_ and various concentrations of DABCO (**left**) and TMPD (**right**). Spectrum of the solutions of individual reactants are shown as dashed red (CHI_3_) or blue (DABCO and TMPD) lines. Inserts: Spectra of the complexes obtained by subtraction of the absorption of components from the spectra of their mixtures.

Although the UV–Vis spectrum of DABCO is transparent at λ > 300 nm, an addition of this amine to an acetonitrile solution containing iodoform resulted in an increase in absorption in the 300–400 nm range. The subtraction of the absorption of each component showed that this increase is related to the appearance of a pair of close absorption bands (Figure 2). In solutions with a constant concentration of iodoform, the intensity of these bands increased with the increase in concentration of DABCO. The addition of DABCO to bromoform also resulted in the appearance of a new absorption band in the UV–Vis spectra, as described earlier [24]. This new band is substantially blue-shifted compared with that observed with CHI_3_, and it was partially overshadowed by the absorption of DABCO itself. In comparison, solutions of DABCO and CHCl_3_ did not show any new absorption beyond 280 nm (spectra of the solutions containing CHCl_3_ and DABCO are the same as the spectra of individual DABCO), and the strong absorption of DABCO hinders measurements below this wavelength.

Similar UV–Vis measurements of interactions of haloforms with TMPD were hindered by the strong absorption of TMPD in the 200–380 nm range. As such, only part of the new absorption of the complex of TMPD with CHI_3_ could be observed at λ > 380 nm, and the corresponding bands of the complexes with CHBr_3_ or with CHCl_3_ are apparently overshadowed by the absorption of TMPD. As such, the spectra of the solutions containing either CHBr_3_ or CHCl_3_ and TMPD are the same as the spectra of individual TMPD. Appearances of new absorptions were also observed upon the addition of the other *N,N*-dimethylanilines or trimethylamine to CHI_3_ (Figures S1–S4 in the Supplementary Materials). A Benesi–Hildebrand treatment [30] of the variations of the absorption intensities with concentrations of amines produced straight lines with R^2^ > 0.99, and the data were also well fit by 1:1 binding isotherms (Figures S5 and S6 in the Supplementary Materials). However, such treatments did not take into account the presence of the two equilibria (Equations (1) and (2)). To further elucidate competitions of XB and HB, we carried out NMR measurements of the analogous solutions.

### 2.2. ^1^H NMR Study of the Interaction of CHX_3_ with Amines

**Figure 3:**
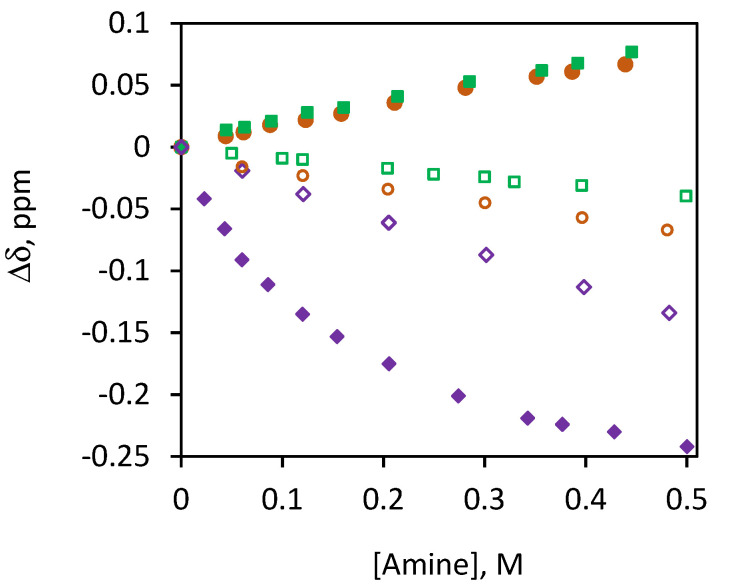
Dependencies of the chemical shifts in the proton of CHI_3_ (◇), CHBr_3_ (ο) and CHCl_3_ (□) (as compared to that in the corresponding isolated molecules) on the concentration of DABCO (filled symbols) or TMPD (open symbols) (in CD_3_CN, 22 °C).

The addition of DABCO to the solution of CHI_3_ in deuterated acetonitrile resulted in the shift in the signal of the haloform’s proton to lower ppm values (Figure 3), indicating an increased shielding of this proton. A similar addition of DABCO to bromoform or chloroform produced a shift in its proton signal to higher ppm values. These results are consistent with earlier studies of the interaction of haloform with halide anions [17], and they suggest a prevalence of XB complexes in solutions of iodoforms with aliphatic amines, and a domination of HB in similar solutions with bromoform or chloroform. The opposite shifts in the proton signals in the XB and HB complexes with DABCO are apparently related to the polarization of haloform by this electron-rich nucleophile. In XB complexes, it results in the shift in electron density from the bonded halogen to the unbonded halogen and hydrogen atoms, increasing the shielding of the latter. In contrast, the polarization of HB complex results in a shift in the electron density from the bonded proton, decreasing its shielding. However, an addition of TMPD to any of the haloforms under study produced a shift to lower ppm values, indicating an increased shielding of this proton (Figure 3). NMR measurements of interactions of haloforms with the other aliphatic and aromatic amines confirm the trends observed with DABCO and TMPD. Specifically, the addition of trimethylamine to CHI_3_ resulted in the shift in the proton signal lower ppm and similar experiments with CHBr_3_ or CHCl_3_ resulted in a shift in the opposite direction. On the other hand, the titrations of any of the haloforms with aromatic amines led to shifts in the proton signals to lower ppm values (Figures S7–S9 in the Supplementary Materials). To clarify the results of the UV–Vis and NMR measurements, we turned to the X-ray structural analysis of the associations and computational analysis of the XB and HB complexes of haloforms with amines.

### 2.3. X-ray Structural Analysis of Co-crystals of Iodoform with TMPD or DABCO

**Figure 4:**
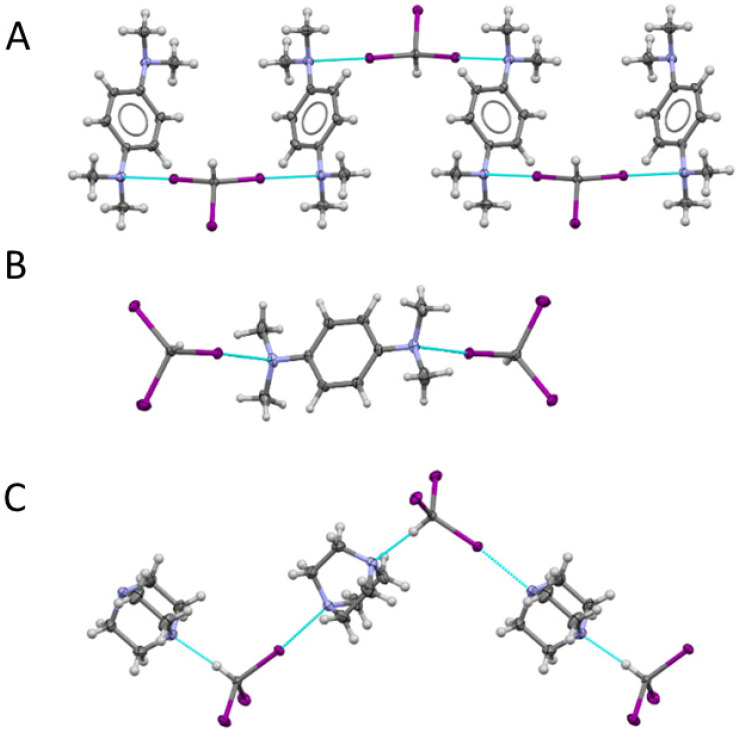
X-ray structures of co-crystals of iodoform with TMPD ((**A**): 1:1, (**B**): 2:1) and DABCO (**C**) showing alternating halogen (and hydrogen)-bonded zigzag chains or discrete 1:2 complexes.

Cooling down acetonitrile solutions containing equimolar quantities of iodoform and either TMPD or DABCO led to formation of co-crystals suitable for X-ray structural measurements. X-ray analysis showed that these co-crystals comprise zigzag chains consisting of alternating iodoform and either TMPD or DABCO molecules (Figure 4A,C). Co-crystallization of CHI_3_ with TMPD also produced discrete 2:1 complexes (Figure 4B).

Chains of TMPD with iodoform in their 1:1 co-crystals were formed by I–N halogen bonding between these molecules involving two iodine substituents of each CHI_3_ and two amino groups of each TMPD. The I–N distances of 2.902 Å were about 22% shorter than the sum of the van der Waals radii of these atoms, and the C–I–N angles were close to linear (177.9 deg), as is typical for halogen bonding. The (centrosymmetric) discrete 2:1 CHI_3_:TMPD complexes also show a pair of halogen bonds with a slightly shorter bond length of 2.842 Å and the C–I–N angles of 172.4 deg. In comparison, DABCO molecules were linked with iodoform by I–N halogen and H–N hydrogen bonding. Both these bonds were close to linear (177.1 deg and 174.2 deg for HB and XB, respectively) and quite short (HB and XB bond length of 2.152 Å and 2.756 Å, respectively). Interestingly, the I–N distances in associations of DABCO with iodoform were shorter than the Br–N distances of 2.877 Å reported earlier [24] in the similar zigzag chains formed by both halogen and hydrogen bonding of this nucleophile with bromoform. This indicates substantially stronger XB involving iodine atoms. Overall, similar to the co-crystals with halide anions, the interaction of haloforms with aromatic or aliphatic amines shows both modes (X–N and H–N) of interactions.

### 2.4. Computational Analysis of XB and HB Complexes

**Figure 5:**
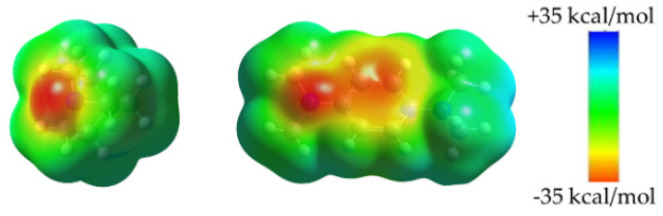
Electrostatic potential (calculated at 0.001 electron bohr^−3^ electronic density) on the molecular surfaces of DABCO (**left**) and TMPD (**right**).

Surface electrostatic potentials of TMPD and DABCO are illustrated in Figure 5. Both these molecules show areas of negative potentials corresponding to the location of lone pairs on the surface of the nitrogen atoms. The magnitude of the minimum (most negative) potential, V_S,min_, of −36.4 kcal/mol on the surface of DABCO is somewhat higher than that on the surface of the nitrogen atom of TMPD (−33.2 kcal/mol), apparently due to the partial delocalization of nitrogen’s lone pairs to the aromatic ring in the latter.

**Figure 6:**
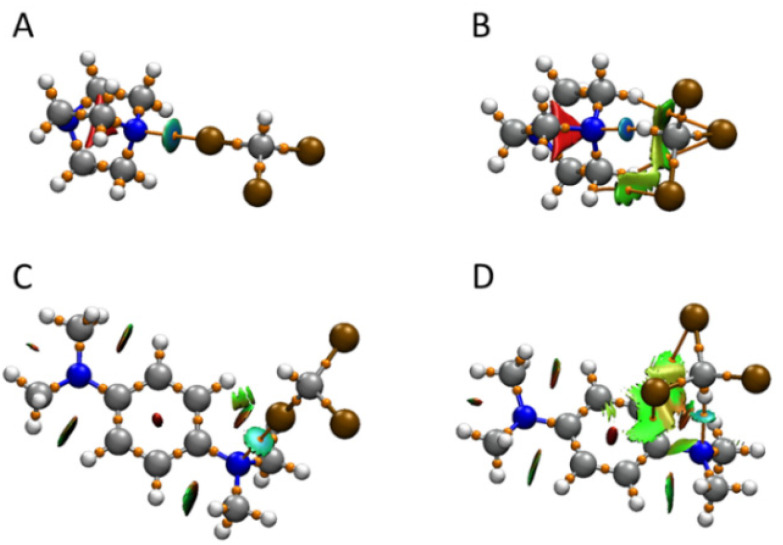
Superposition of the results QTAIM and NCI analyses onto the structures of the XB complexes of CHBr_3_ with DABCO (**A**) and TMPD (**C**) and HB complexes with DABCO (**B**) and TMPD (**D**). The bond paths and critical (3,−1) points (from QTAIM) are shown as orange lines and spheres, respectively, and blue-green discs indicate areas of bonding interactions (from NCI).

For the TMPD molecule, the negative potential is extended from the surface of the nitrogen atom to the aromatic ring. The center of the aromatic ring shows another minimum potential of about −30 kcal/mol. The locations of V_S,min_ on the surfaces of nitrogen atoms in TMPD and DABCO suggest they would be attracted to *σ*-holes on the surface of either hydrogen or halogen atoms in haloforms. Indeed, DFT M062X/def2tzvpp calculations (see Experimental for details) produced energy minima corresponding to XB and HB complexes between all three haloforms and nitrogen atoms of aromatic or aliphatic amines. The structural features of halogen (and, for the complexes with DABCO, hydrogen bonds found for these minima (illustrated in Figure 6) were consistent with the geometries obtained via X-ray crystallographic analysis of the solid-state associations. (In addition, calculations of complexes with TMPD produced minima in which hydrogen or halogen substituents of haloforms were directed toward carbon atoms in an aromatic ring or the middle of C–N bonds; vide infra.

Quantum Theory of Atoms in Molecules (QTAIM) analysis [31] of the optimized structures showed bond paths (orange lines in Figure 6) from nitrogen atoms to halogen or hydrogen substituents of haloforms in XB and HB complexes, respectively. It also revealed (3,−1) bond-critical points (BCPs) along these bond paths (small orange spheres). Bonding interactions between nucleophilic nitrogen atoms and halogen or hydrogen of haloform were further confirmed by the non-covalent index (NCI) analysis [32]. The NCI treatment showed the presence of the blue-colored discs located at the BCPs between nitrogen atoms of amines and halogen or hydrogen atoms of haloforms, which indicates moderately strong intermolecular attraction between these atoms. It should be mentioned that besides the bond paths and BCPs between nitrogen atoms and hydrogens, HB complexes showed bond paths and BCPs between halogen atoms of haloforms and hydrogen substituents or aromatic carbons of amines. The NCI analysis showed green surfaces corresponding to non-bonding or very weak bonding interaction along these bond paths. This indicates that they represent secondary interactions most likely supported by the close approach of haloform to amines. Interaction energies, XB and HB lengths and C–I–N or C–H–N angles for the representative complexes are listed in Table 1.

The characteristics of the BCPs on the XB and HB bond paths obtained from the QTAIM analysis corroborate the similarities of these associations between amines and haloforms. The electron densities and energies at these BCPs are listed in Table 2.

TD DFT calculations showed that UV–Vis spectra of all XB complexes contain absorption bands (Table 3) which are red-shifted and substantially more intense than the absorption bands in the individual compounds. These bands are related to the transition involving orbitals localized on both haloform and amines. On the other hand, UV–Vis spectra of the optimized XB complexes are very close to that of the superposition of individual components. These results are consistent with the reported data, and they indicate that the appearance of new absorption bands in the UV–Vis range is related to the formation of XB complexes.

**Figure 7:**
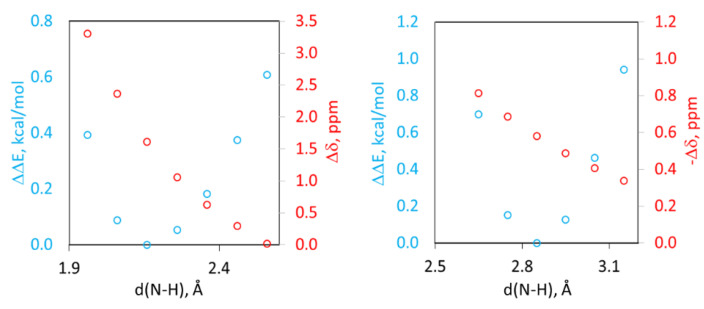
Dependencies of the energies (blue circles) of HB (**left**) and XB (**right**) complexes relative to that of the energy minimum and the chemical shifts in the proton (red circles) relative to that in the individual CHI_3_ on interatomic separations.

The proton signals of haloforms in the NMR spectra of the optimized XB complexes were shifted to lower ppm values indicating increased shielding of these protons. In the case of all HB associations, the signals were shifted to higher ppm values. These results agree with earlier observations of opposite shifts in the haloforms’ proton signals related to halogen and hydrogen bonding [14,15,16,17]. However, despite the fact that calculations (and the data on similar associations) suggest that CHBr_3_ and especially CHCl_3_ form stronger HB complexes with TMPD, experimental measurements showed a uniform shift in the haloforms’ proton signal to lower ppm values upon addition of this amine to any of the haloforms (Figure 3). It should be noted, however, that in contrast to the singular solid-state donor/acceptor arrangement, solution-phase complexes are subject to fluctuations around the optimized minimum (or several local minima) which might affect spectral characteristics. Indeed, an analysis of the potential energy landscape shows that the XB and HB complexes between CHX_3_ and TMPD are characterized by a shallow minimum. The variations of the X–N or H–N separations by about 0.5 Å are accompanied by energy changes of less than 1 kcal/mol (Figure 7, note that complexes of CHBr_3_ and CHCl_3_ with TMPD, and associations of haloforms with DABCO showed similar shallow minima; see Figure S10 in the Supplementary Materials).

**Figure 8:**
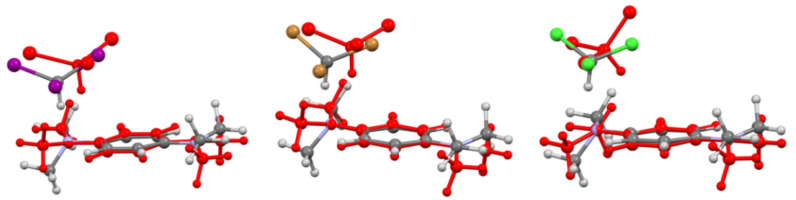
Structural overlap of the associations between TMPD and CHI_3_ (**left**), CHBr_3_ (**middle**) and CHCl_3_ (**right**) formed via H–N bonding and alternative complexes (shown as red structures).

These structural variations are accompanied by changes in NMR spectra, i.e., the increase in the separation is accompanied by a decrease in the difference in the position of the signal in the complex compared with that of the individual haloform. While shallow minima imply the co-existence of assemblies of associations with varying separations, the average distances and NMR shifts for these assemblies seem to be close to those found for the minimum. As such, they would not substantially affect general trends of the NMR shifts. However, the analysis of the potential energy landscape also revealed the presence of the additional minima for the complexes of haloforms with TMPD. Such minima are apparently related to the presence of additional binding sites on the surface of TMPD due increased electron density on the aromatic ring, as shown in Figure 5). The structural overlap (Figure 8) demonstrates that the alternative HB structures were quite similar. The main structural difference was the shift in the position of the protons in the alternative structures from the nitrogen atom toward the aromatic ring, so it was directed toward the middle of the C–N bond or toward the aromatic carbon in ortho-position with respect to the amino group. The differences in energies of these alternative structures and the corresponding minima showing hydrogen bonding with nitrogen atoms were 0.9 kcal/mol, 1.1 kcal/mol and −0.1 kcal/mol for associations with CHI_3_, CHBr_3_ and CHCl_3_, respectively. Despite such seemingly minor structural and thermodynamic differences, the proton signal in the NMR spectra of the haloform in the alternative complexes were shifted to lower ppm values by 1.41 ppm, 1.21 ppm and 0.72 ppm in complexes of TMPD with CHI_3_, CHBr_3_ and CHCl_3_, respectively, compared with the signal of the individual haloform molecules.

Computational analysis also revealed the presence of the XB complexes with TMPD in which the halogen atom is directed toward aromatic carbons. Specifically, in the alternative structure of CHI_3_ with this molecule, the iodine substituent of iodoform is directed toward a nitrogen-bonded carbon atom (Figure S11 in the Supplementary Materials). The energy of this structure was about 2.0 kcal/mol higher than that of the complex with the I–N bond. Most notably, similarly to the structure with the I–N interaction, the iodoform proton in the alternative XB structure was shifted by about 0.55 ppm to lower ppm values, and its UV–Vis spectrum contained a new absorption band with λ_max_ = 392 nm, ε = 2900 M^−1^ cm^−1^.

### 2.5. Unified Correlation of Strength of the XB and HB Complexes with the Surface Electrostatic Potentials in the Polarized Molecules

The differentiation and simultaneous measurements of XB and HB complexes which are formed by the same pairs of molecules in solutions are challenging tasks that require an accurate knowledge of the distinctions between these two interactions.

**Figure 9:**
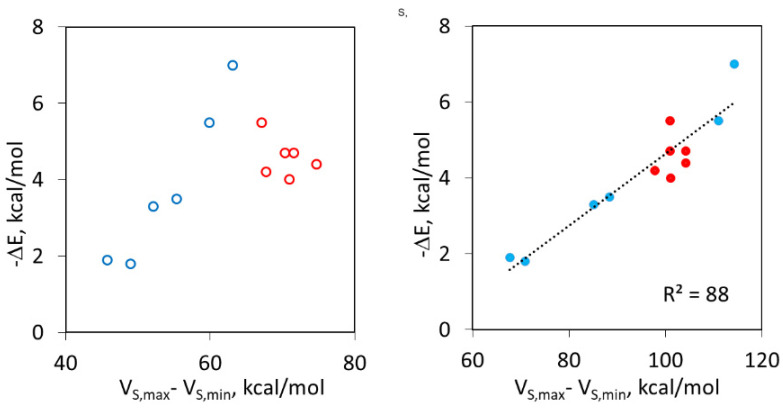
Correlations between the interaction energies in the XB and HB complexes (blue and red circles, respectively) with DABCO and TMPD and the difference of the electrostatic potentials on the surfaces of interacting atoms in the individual molecules (**left**) and in polarized molecules (**right**).

In accordance with the results of the X-ray crystallographic analysis, computations of complexes of haloforms with DABCO or TMPD produced energy minima showing I–N or H–N bonding. The data in Table 1 show that XB strength between CHX_3_ and amines decreased in the expected order for this interaction with X as I > Br > Cl. For iodoform, this interaction was somewhat stronger in XB complexes with DABCO than that with TMPD. These computational results agree with the experimental X-ray structural data, i.e., shorter I–N separations in the solid-state complexes of CHI_3_ with DABCO than that in associations with TMPD, and shorter I–N distances in complexes of iodoform with DABCO than Br–N distances reported in the similar associations with CHBr_3_. The differences in the ΔE values for complexes of these aromatic and aliphatic amines with either bromoform or chloroform were small, if any. The HB complexes of iodoform with amines were also slightly stronger than with those with bromoform and chloroform, and all energies were within a −5 ± 1 kcal/mol range. To clarify the reasons for the variations of the interaction energies, we compared their values with the changes in the maximum (V_S,max_) and minimum (V_S,min_) electrostatic potentials on the surfaces of haloforms and amines, respectively [34]. The dependence of the ΔE values on the difference V_S,max_ − V_S,min_ (found for the individual haloforms and amines) is shown as open circles in Figure 9, left (red and blue colors denote HB and XB complexes, respectively).

For the XB complexes, the increase in the difference of potentials is accompanied by an increase in the magnitude of (negative) ΔE values. For the HB complexes, however, no such correlation was observed. Furthermore, while the V_S,max_ values on the surfaces of hydrogens atoms are higher than those on the surfaces of halogen in all individual haloforms (which is reflected in the V_S,max_ − V_S,min_ differences), halogen bonding is the dominant mode of interaction of iodoform. As such, the overall R^2^ value for the whole set of XB and HB complexes is just 0.33.

It should be noted, however, that the presence of the electron-rich species near the haloforms may substantially affect electron distributions in these species (and the same is true for the amines). Such polarization represents an important factor in the strength of intermolecular complexes [35]. Thus, we evaluated electrostatic potentials on the surfaces of haloforms in the presence of the partial charge located at the positions of the bonded nitrogen atoms in the HB and XB complexes (see Experimental for details). The values of V_S,max_ on the surface of halogen and hydrogen atoms in the presence of the charges are substantially higher than those of the individual molecules (see Table S1 in the Supplementary Materials). The corresponding correlation between ΔE and V_S,max_ − V_S,min_ values calculated using V_S,max_ in the polarized CHX_3_ molecules is shown in Figure 9 (right) as the filled circles. While this approach takes into account only the polarization of haloforms, it considerably improves the correlation. The points corresponding to the HB complexes follow the same trend line as the XB associations (with R^2^ = 0.88 for the whole series). This indicates that once polarization is taken into account, the strengths of the HB and XB complexes between haloforms and aromatic and aliphatic amines can be uniformly related to the electrostatic potentials on the surfaces of HB/XB donors and acceptors.

The variations in the electron densities and energies at BCPs (which are most commonly used for characterization of bonding strength and nature [36]) for different complexes in Table 2 follow the trends observed in energies and bond lengths listed in Table 1. In particular, electron densities at the BCPs for XB complexes of CHI_3_ are higher than that of the corresponding HB associations, and the values in the range 0.02–0.04 a.u. are consistent with the strong intermolecular bonding in these complexes [36,37]. For the XB complexes, ρ(r) values decrease from complexes of CHI_3_ to those of CHBr_3_ and CHCl_3_; however, the values for the XB complexes are rather uniform. As such, the relative values of electron density for the HB complexes of chloroform and bromoform are higher than those for the XB associations. Very small negative or positive values of the energy density H(r) for the complexes in Table 2 are also consistent with the strong intermolecular interactions [36,37], and their variations are consistent with the changes in ΔE.

### 2.6. Differentiation of XB and HB Complexes Based on Their UV–Vis and NMR Characteristics

**Figure 10:**
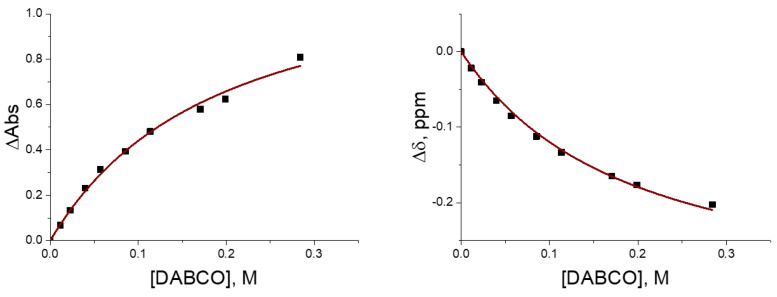
Dependencies of the ΔAbs and Δδ values in the solutions with constant concentration of CHI_3_ (0.01 M) and variable concentrations of DABCO. Solid lines show the simultaneous multivariable fitting of the UV–Vis and NMR titrations data.

While bonding characteristics of the optimized HB and XB complexes were quite similar, the UV–Vis and NMR spectral characteristics of these associations were different. Most notably, formation of the XB complexes is accompanied by the appearance of new intense absorption bands in the UV–Vis spectra of the solutions containing haloforms and amines (although they were overshadowed by the absorption of their components for some systems). In comparison, the electronic spectra of HB complexes are close to the superposition of the spectra of the individual reactants. Additionally, halogen and hydrogen bonding of CHX_3_ with aliphatic amines led to the shift in the NMR signals of haloforms in opposite directions, i.e., halogen bonding resulted in the signal shift to the lower ppm values, and hydrogen bonding led to the shift to the higher values. These data were consistent with earlier studies of the interactions of haloforms with the other nucleophiles [14,15,16,17,18]. Thus, the multivariable analysis of the data obtained from the UV–Vis and NMR measurements (as described in our previous work [14]) of the solutions containing constant concentrations of haloforms and variable concentrations DABCO (Figure 10) allowed us to evaluate equilibria constant for the XB and HB complexes co-existing in solutions. For the DABCO complexes with CHI_3_, this treatment produced values of K_X_ = 3.7± 0.3 M^−1^ and K_H_ = 2.0 ± 0.2 M^−1^ and the formation constants for associations with CHBr_3_ were K_X_ = 0.27 ± 0.03 M^−1^ and K_H_ = 0.12 ± 0.01 M^−1^.

The differentiation of the effects of HB and XB with aromatic amines using NMR data is complicated by the possible presence of the XB complexes with the rather small differences in energies, but opposite directions of shifts in proton signals (compared with those in the individual molecules). As such, the values of these constants were estimated using UV–Vis spectral data which reflected formation of XB associations Assuming that the ratio K_H_/K_X_ = exp (ΔΔE/RT) ≈ 1 (where ΔΔE = 0 is a difference of interaction energy of XB and the most stable HB complex for CHI_3_/TMPD pair, see Table 1), the values of both formation constants are roughly 0.3 M^−1^.

## 3. Materials and Methods

Commercially available haloforms, TMPD and DABCO, were purified by distillation or sublimations.

The UV–Vis measurements were carried out on a Cary 5000 spectrophotometer (Agilent, Santa Clara, CA, USA) in dry (HPLC grade) acetonitrile. NMR measurements were performed on a 400 MHz spectrometer Jeol 400 (Jeol USA Inc., Peabody, MA, USA) in deuterated acetonitrile with internal TMS standard. The intensities of the absorption of [CHX_3_, D] complexes, ΔAbs, were obtained by the subtraction of the absorption of the components from the spectrum of the mixtures of CHX_3_ and amine. The K_XB_ and K_HB_ values were obtained by the simultaneous nonlinear fitting (using the multiple variable option with Levenberg–Marquardt iteration algorithm in OriginPro 2016) of the dependencies of ΔAbs and Δδ on the concentrations of amines measured at the same concentrations of CHX_3_ using Equations (3) and (4), as described in detail earlier [14]:

ΔAbs = ε*l* × C_XB_ = ε*l* × {(C^o^_A_ + C^o^_D_ + 1/(K_XB_ + K_HB_)) − ((C^o^_A_ + C^o^_D_ + 1/(K_XB_ + K_HB_))^2^ − 4C^o^_A_C^o^_D_)^0.5^}/(2 (1 + K_HB_/K_XB_)) (3)

Δδ = Δδ_XB_/C^o^_D_ × C_XB_ + Δδ_HB_/C^o^_D_ × C_HB_ = [Δδ_XB_/C^o^_D_ × {(C^o^_A_ + C^o^_D_ + 1/(K_XB_ + K_HB_)) − ((C^o^_A_+C^o^_D_ + 1/(K_XB_ + K_HB_))^2^−4C^o^_A_C^o^_D_)^0.5^} + Δδ_HB_/C^o^_D_ × {(C^o^_A_ + C^o^_D_ + 1/(K_XB_ + K_HB_)) − ((C^o^_A_ + C^o^_D_ + 1/(K_XB_ + K_HB_))^2^ − 4C^o^_A_C^o^_D_)^0.5^}]/(2 (1 + K_XB_/K_HB_)) (4)

where C_com_ is the concentration of the complex, and C^o^_D_ and C^o^_A_ are initial concentrations of CHX_3_ and amine, ε and *l* are extinction coefficient of the complex and the length of the cell which was used in the UV–Vis measurements, and Δδ_∞_ = δ_∞_ − δ_0_ is the difference between the ppm of the CHX_3_ proton in the presence of an infinite concentration of amine, δ_∞_ (obtained from the calculations of these complexes) and that of the separate CHX_3_, δ_0_ and K_XB_ and K_HB_ are formation constants of the XB and HB complexes.

Geometries of the XB and HB complexes and their components were optimized without constraints in acetonitrile via M06-2X/def2tzvpp calculations (with a polarizable continuum model) using the Gaussian 09 suite of programs [38,39,40]. The interaction energies were determined as: ΔE = E_comp_ − (E_CHX3_ + E_A_) + BSSE, where E_comp_, E_CHX3_ and E_A_ are sums of the electronic and ZPE of the complex, CHX_3_ and amine (DABCO or TMPD) and BSSE is a basis set superposition error [41]. UV–Vis spectra of complexes and trihalomethanes were calculated via TD-DFT calculations, proton NMR shifts were obtained via GIAO calculations using geometries of the complexes optimized in acetonitrile. Molecular electrostatic potentials of the polarized molecules were calculated by placing point charges corresponding to the charge of nitrogen in DABCO (calculated as ESP charges) at the position where such atoms are located in the optimized complexes. Such approximation allowed to take into account various polarizabilities of interacting atoms in XB and HB complexes and led to reasonable correlation between electrostatic potentials and interaction energies. QTAIM and NCI analyses were performed with Multiwfn [42] using wfn files generated by Gaussian 09. The results were visualized using the molecular graphics program VMD [43]. Details of the calculations, energies, geometric and spectral characteristics of HB and XB complexes as well as atomic coordinates of the calculated complexes are listed in the ESI.

The single crystals were measured on a Bruker Quest diffractometer (Bruker AXS, LLC, Madison, WI, USA) with a fixed chi angle, a sealed tube fine focus X-ray tube, single crystal curved graphite incident beam monochromator (Bruker AXS, LLC, Madison, WI, USA), a Photon100 area detector (Bruker AXS, LLC, Madison, WI, USA) and an Oxford Cryosystems low-temperature device (Hanborough House, Oxford, United Kingdom). Examination and data collection were performed with Mo Kα radiation (λ = 0.71073 Å). Reflections were indexed and processed, and the files were scaled and corrected for absorption using APEX3 [44]. The space groups were assigned, and the structures were solved by direct methods using XPREP within the SHELXTL suite of programs [45] and refined by full matrix least squares against F^2^ with all reflections using Shelxl2018 [46,47] using the graphical interface Shelxle [46]. If not specified otherwise, H atoms attached to carbon and nitrogen atoms were positioned geometrically and constrained to ride on their parent atoms, with C–H bond distances of 1.00, 0.99 and 0.98 Å for aliphatic C–H, CH_2_ and CH_3_ moieties, respectively. Methyl H atoms were allowed to rotate but not to tip to best fit the experimental electron density. U_iso_(H) values were set to a multiple of U_eq_(C) with 1.5 for CH_3_, and 1.2 for C–H units, respectively. Crystallographic, data collection and refinement details are listed in Table S2 in the Supplementary Materials. Complete crystallographic data, in CIF format, have been deposited with the Cambridge Crystallographic Data Centre. CCDC 2202934, 2202935 and 2206546 contain the supplementary crystallographic data for this paper. These data can be obtained free of charge via www.ccdc.cam.ac.uk/data_request/cif.

## 4. Conclusions

Experimental and computational analysis of interactions of haloforms with aromatic and aliphatic amines highlighted the similarities and distinctions of HB and XB complexes, which will be helpful for the identification and quantitative characterization of these competing interactions in chemical and biochemical systems. We demonstrated that when polarization of haloforms is taken into account, the interaction energies within the HB and XB complexes of CHX_3_ molecules with TMPD and DABCO follow the same correlation with the difference electrostatic potentials on the surfaces of the interacting atoms. The electron densities and energies at BCPs along the H–N and N–X bond paths also follow the same trends. These data confirm that the thermodynamics of these moderately strong associations is dominated by electrostatic interactions. However, spectral properties of the XB and HB complexes were quite different. The most consistent distinction is observed in the UV–Vis spectra of the complexes. Halogen bonding is accompanied by an appearance of strong absorption bands related to the formation of the XB associations (which suggests substantial molecular-orbital interactions between haloform and amine within these complexes). In contrast, the spectral of the HB associations were close to the superposition of the spectra of the individual reactants. The effects of intermolecular interactions on the NMR spectra were dependent on the nature of the amine. In particular, the HB and XB associations of haloforms with aliphatic amines led to the opposite shifts in their protons’ signals in the NMR spectra. Thus, combination of the UV–Vis and NMR data allows to differentiate XB and HB complexes of haloforms with these amines in solutions. XB with aromatic amines led to the shift in the same direction as the aliphatic ones; however, the corresponding effects of HB of CHX_3_ with aromatic amines were complicated by the presence of multiple HB minima in which hydrogens were directed either toward the nitrogen atom or C–N bond or aromatic carbon. The haloforms’ protons signals in the NMR spectra of these complexes were shifted in the opposite direction, which hinder the application of this method for quantitative analysis of XB and HB complexes.

## Figures and Tables

**Table 1:** Interaction energies and interatomic distances in the XB and HB complexes.

CHX_3_	B	XB Complexes			HB Complexes		
		ΔE, kcal/mol	d_X-N_, Å	R_XN_^a^	ΔE, kcal/mol	d_H-N_, Å	R_HN_^a^
CHI_3_	DABCO	−7.0	2.694	0.72	−4.7	2.051	0.75
	TMPD	−5.5	2.845	0.76	−5.5	2.163	0.79
CHBr_3_	DABCO	−3.5	2.802	0.79	−4.4	2.043	0.78
	TMPD	−3.3	2.872	0.81	−4.7	2.095	0.76
CHCl_3_	DABCO	−1.8	2.931	0.89	−4.0	2.095	0.76
	TMPD	−1.9	2.892	0.88	−4.2	2.183	0.79

^a^ Normalized interatomic separations R_XN_ = d_X⋯N/_(r_X_ + r_N_), where r_X_ and r_N_ are van der Waals radii [33].

**Table 2:** Electron densities and energies (ρ(r) and H(r), in a.u.) at BCPs along XB and HB bond paths.

CHX_3_	B	XB Complexes		HB Complexes	
		ρ(r) × 10^2^	H(r) × 10^3^	ρ(r) × 10^2^	H(r) × 10^3^
CHI_3_	DABCO	3.65	−3.70	2.76	−1.11
	TMPD	2.70	−1.11	2.15	0.44
CHBr_3_	DABCO	2.39	0.51	2.81	−1.31
	TMPD	2.07	0.76	2.16	0.34
CHCl_3_	DABCO	1.52	1.61	2.53	−0.37
	TMPD	1.66	1.51	2.05	0.57

**Table 3:** Calculated UV–Vis and NMR characteristics of the XB and HB complexes ^a^.

CHX_3_	B	XB Complexes			HB Complexes		
		λ_max_, nm	ε × 10^−3^, M^−1^ cm^−1^	Δδ, ppm ^b^	λ_max_, nm	ε × 10^−3^, M^−1^cm^−1^	Δδ, ppm ^b^
CHI_3_	DABCO	311	9.0	−1.539	302	1.4	1.847
	TMPD	335	7.2	−0.669	295	4.5	1.506
CHBr_3_	DABCO	259	7.5	−0.452	212	2.8	2.502
	TMPD	285	8.0	−0.437	245	20.0	1.503
CHCl_3_	DABCO	216	3.3	−0.230	220	250	2.119
	TMPD	290	3.0	−0.344	247	20.1	1.353

^a^ λ, in nm, (ε) for individual compounds are: CHI_3_: 304 (3050), CHBr_3_: 223 (2500), CHCl_3_: 175 (1600), DABCO-TMPD 245 (24,400), δ (in ppm) for CHI_3_: 7.0287, CHBr_3_: 7.6825, CHCl_3_: 7.582. ^b^ Relative to the protons’ chemical shifts in the individual haloforms.

## Data Availability

Crystallographic data have been deposited with the Cambridge Crystallographic Data Centre and can be obtained free of charge (see above). Atomic coordinates and energies of the calculated complexes are available in the Supplementary Information.

## Supplementary Materials

The following supporting information can be downloaded at: https://www.mdpi.com/article/10.3390/molecules27186124/s1. Figure S1: UV–Vis spectra of the solutions of CHI_3_ and 4-methoxy-N,N-dimethylaniline in CH_3_CN. Figure S2: UV–Vis spectra of the solutions of CHI_3_ and 3-methoxy-N,N-dimethylaniline in CH_3_CN. Figure S3: UV–Vis spectra of the solutions of CHI_3_ and 4-(dimethylamino)benzonitrile in CH_3_CN. Figure S4: UV–Vis spectra of the solutions of CHI_3_ and 3-(dimethylamino)benzonitrile in CH_3_CN. Figure S5: Benesi–Hildebrand plots based on the UV–Vis spectra of solutions of CHI_3_ with TMPD and DABCO. Figure S6: Fit of spectral changes in solutions of CHI_3_ with TMPD and DABCO to 1:1 binding isotherm. Figure S7: Dependencies of the chemical shifts in the protons of CHI_3_ on the concentration of added trimethylamine, *N,N*-dimethylaniline, 4-(dimethylamino)benzonitrile, *p*-bromo-*N,N*-dimethylaniline or 3-(dimethylamino)benzonitrile. Figure S8: Dependencies of the chemical shifts in the protons of CHBr_3_ on the concentration of added trimethylamine, *N,N*-dimethylaniline, 4-(dimethylamino)benzonitrile, *p*-bromo-*N,N*-dimethylaniline or 3-(dimethylamino)benzonitrile. Figure S9: Dependencies of the chemical shifts in the protons of CHBr_3_ on the concentration of added trimethylamine, *N,N*-dimethylaniline, 4-(dimethylamino)benzonitrile, *p*-bromo-*N,N*-dimethylaniline or 3-(dimethylamino)benzonitrile. Figure S10. Effect of variation of interatomic H–N and X–N separations on the energy of HB and XB complexes and chemical shifts in the haloforms’ protons. Figure S11. Alternative structure of XB complex between CHI_3_ and TMPD. Table S1: Values of the maximum electrostatic potentials on the surfaces of halogen and hydrogen atoms in individual and polarized haloforms. Table S2. Crystallographic, data collection and refinement details. Table S3: Energies of the HB and XB complexes and their components. Table S4: Atomic coordinate of the HB and XB complexes of haloforms with DABCO and TMPD.

## Sample Availability

Substances used in the study are commercially available and they are not available from the authors.
