# Supplementary material for: Thermodynamics and Spectroscopy of Halogen- and Hydrogen-Bonded Complexes of Haloforms with Aromatic and Aliphatic Amines
Source: Molecules. 2022 Sep 19;27(18):6124. doi: 10.3390/molecules27186124 (PMC9500756; doi:10.3390/molecules27186124)
Supplement: Supplementary file 1 [file molecules-27-06124-s001.zip › molecules-1910962-supplementary.pdf]

Supporting Information

for

Thermodynamics and spectroscopy of halogen- and hydrogen-bonded complexes of haloforms with aromatic and aliphatic amines

Emmanuel Adeniyi,[1] Olivia Grounds,[1] Zachary Stephens,[1] Matthias Zeller,[2] and Sergiy V. Rosokha[1]*

[1]*Department of Chemistry, Ball State University, Muncie, IN, 47306, USA*
[2]*Department of Chemistry, Purdue University West Lafayette, IN, 47907, USA*

* Correspondence: svrosokha@bsu.edu

Content

Figure S1: UV-Vis spectra of the solutions of CHI_3 and 4-methoxy-N,N-dimethylaniline.
Figure S2: UV-Vis spectra of the solutions of CHI_3 and 3-methoxy-N,N-dimethylaniline.
Figure S3: UV-Vis spectra of the solutions of CHI3 and 4-(dimethylamino)benzonitrile.
Figure S4: UV-Vis spectra of the solutions of CHI3 and 3-(dimethylamino)benzonitrile.
Figure S5**:** Benesi-Hildebrand plots based on the UV-Vis spectra of solutions of CHI3 with TMPD and DABCO.
Figure S6: Fit of spectral changes in solutions of CHI3 with TMPD and DABCO to 1:1 binding isotherm.
Figure S7: Dependencies of the chemical shifts of the protons of CHI_3 on the concentration of trimethylamine (TEA), N,N-dimethylaniline (DMA), 4-(dimethylamino)benzonitrile (DMABN), p-bromo-N,N-dimethylaniline (BrDMA) or 3-(N,N-dimethylamino)anisidine (MeODMA).
Figure S8: Dependencies of the chemical shifts of the protons of $CHBr_3$ on the concentration of TEA, DMA, DMABN, BrDMA amd MeODMA.
Figure S9: Dependencies of the chemical shifts of the protons of $CHCl_3$ on the concentration of TEA, DMA, DMABN, BrDMA amd MeODMA.
Figure S10. Effect of variation of interatomic H…N and X…N separations on the energy of HyB and HaB complexes and chemical shifts of the haloforms' protons.
Figure S11. Alternative structure of HaB complex between CHI_3 and TMPD.
Table S1. Values of V_{max} on the surfaces of individual and polarized haloforms.
Table S2. Crystallographic, data collection and refinement details.
Table S3: Energies of the HyB and HaB complexes and their components.
Table S4. Atomic coordinate of the HYB and HaB complexes of haloforms with DABCO and TMPD

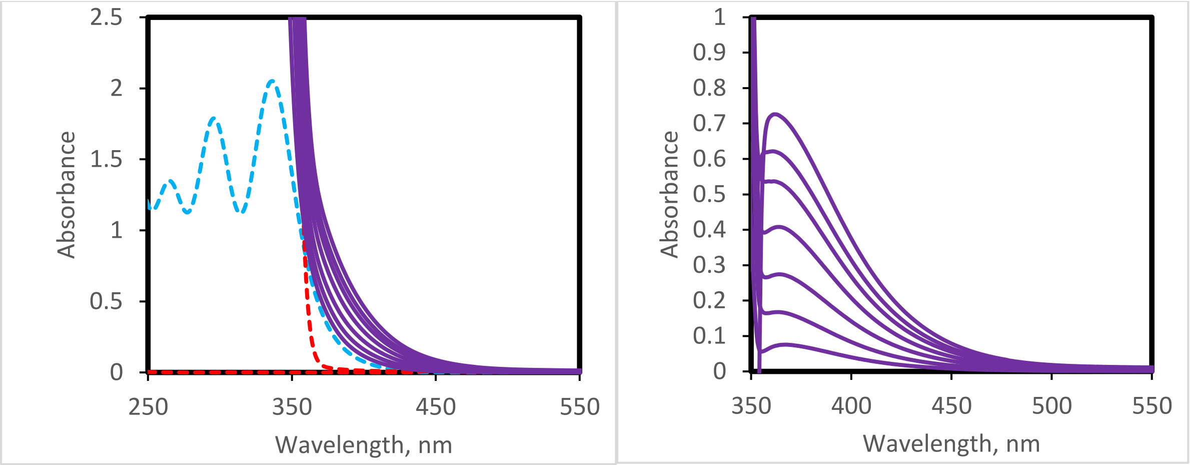

Figure S1. (Left) Spectra of the solutions with constant concentration of CHI_3 and various concentrations of p-methoxy-N,N-dimethylaniline (pMeODMA). Spectra of the solutions of individual reactants are shown as dashed blue (CHI_3) or red (pMeODMA) lines. (Right): Spectra of the complexes obtained by subtraction of the absorption of components from the spectra of their mixtures

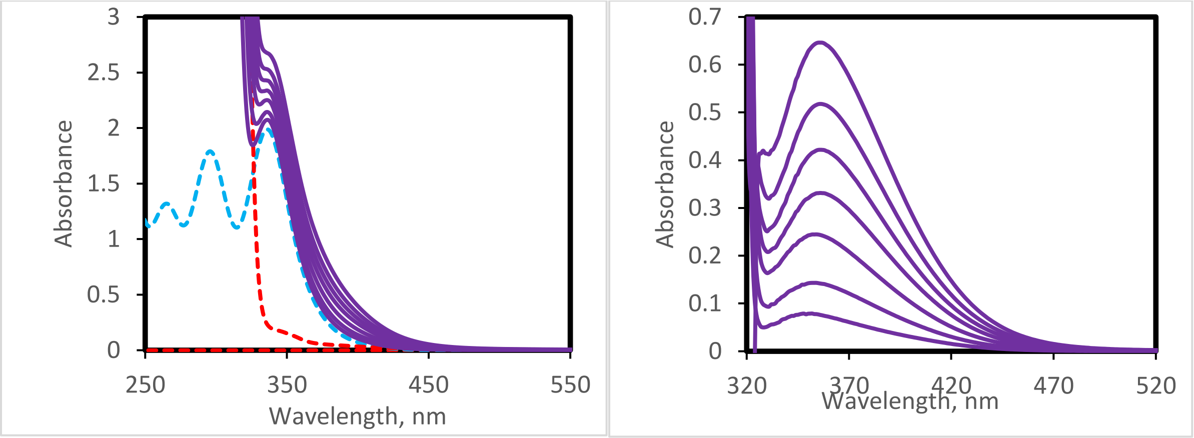

Figure S2. (Left) Spectra of the solutions with constant concentration of CHI_3 and various concentrations of m-methoxy-N,N-dimethylaniline (mMeODMA). Spectra of the solutions of individual reactants are shown as dashed blue (CHI_3) or red (mMeODMA) lines. (Right): Spectra of the complexes obtained by subtraction of the absorption of components from the spectra of their mixtures

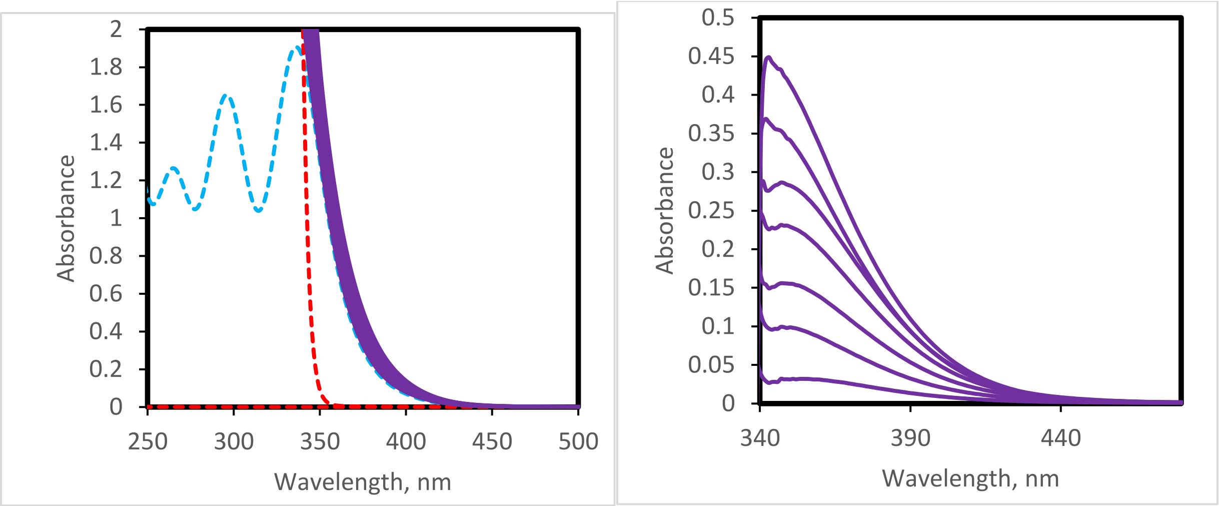

Figure S3. (Left) Spectra of the solutions with constant concentration of CHI_3 and various concentrations of p-cyano-N,N-dimethylaniline (DMACN). Spectra of the solutions of individual reactants are shown as dashed blue (CHI_3) or red (DMACN) lines. (Right): Spectra of the complexes obtained by subtraction of the absorption of components from the spectra of their mixtures

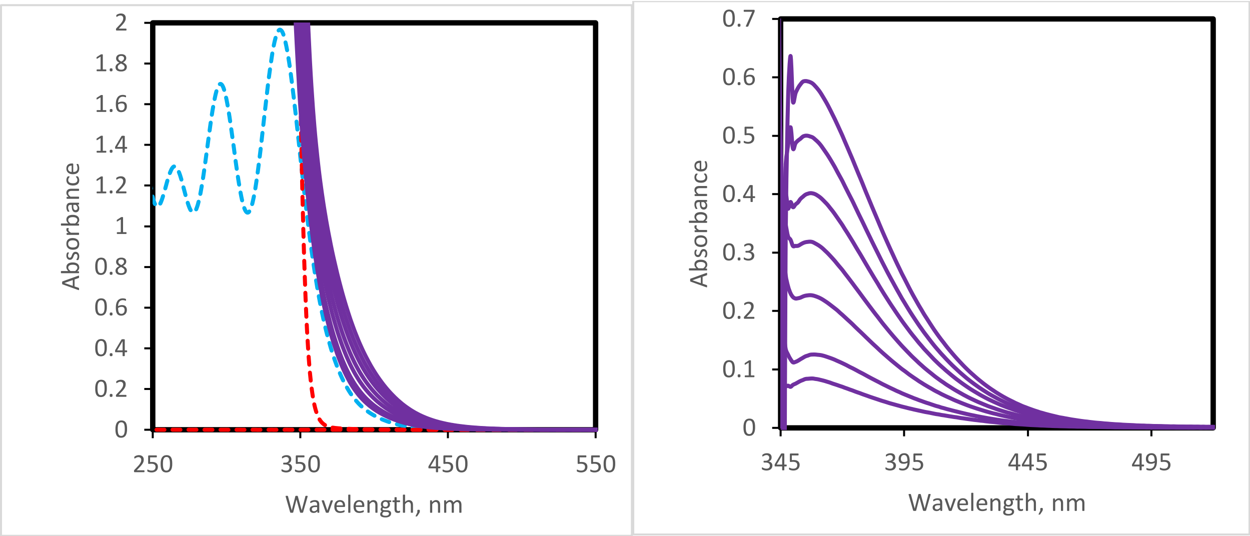

Figure S4. (Left) Spectra of the solutions with constant concentration of CHI_3 and various concentrations of p-bromo-N,N-dimethylaniline (DMABr). Spectra of the solutions of individual reactants are shown as dashed blue (CHI_3) or red (DMABr) lines. (Right): Spectra of the complexes obtained by subtraction of the absorption of components from the spectra of their mixtures

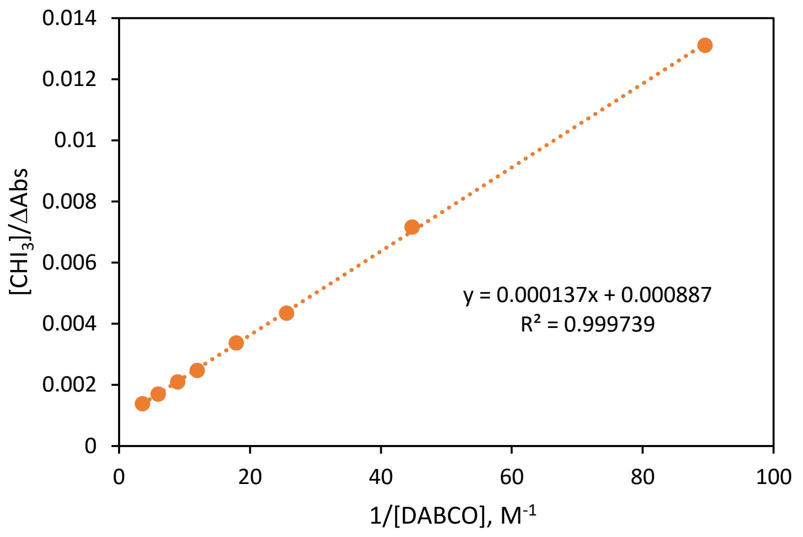

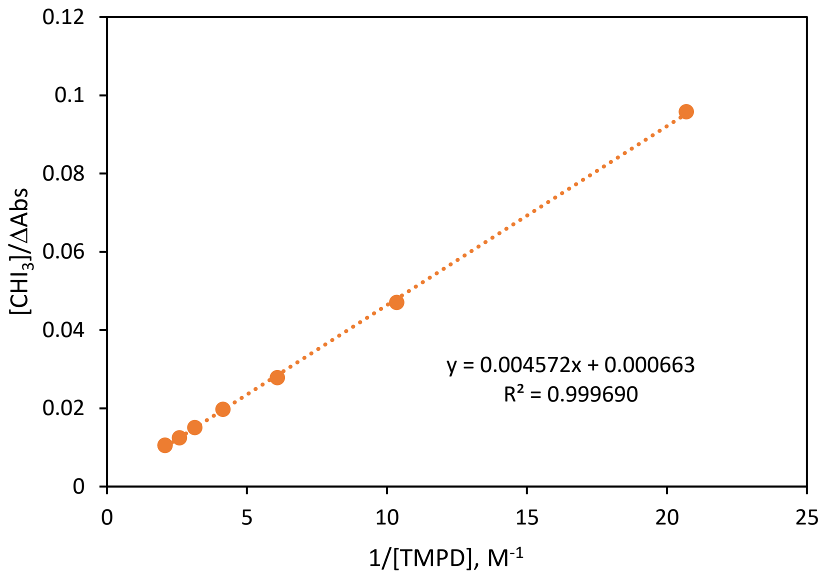

Figure S5. Benesi-Hilderbrandt plots C^o_D /Abs = $1/(\varepsilon\ l)$ + $\{1/(K^{eff}\ \varepsilon l)\} \times 1/[C^o_A]$ (where C^o_D and C^o_A are initial concentrations of CHX_3 and amine, respectively, ε and *l* are extinction coefficient of the complex and the length of the cell which was used in the UV-Vis measurements, K_{eff} is the effective formation constant of the complex and ΔAbs is the absorbance of the complex at certain wavelength (obtained by subtraction of the absorption of the components from the spectra of the mixtures), based on the treatments of spectra measured in solution of CHI_3 with DABCO (top) and TMPD (bottom).

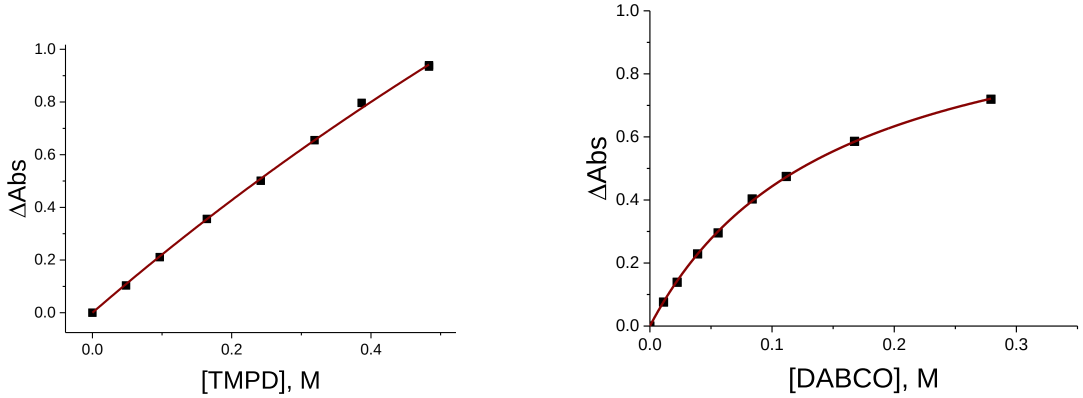

Figure S6. The fit (using Origin 16) of the UV-Vis spectral data of the solutions containing constant concentrations of CHI_3 and variable concentrations of TMPD (left) or DABCO (right) to 1:1 binding isotherm as $\Delta Abs = \varepsilon l \times C_{com} = \varepsilon l \times \{(C^o{}_A + C^o{}_D + 1/K_{eff}) - ((C^o{}_A + C^o{}_D + 1/K_{eff})^2 - 4C^o{}_A C^o{}_D)^{0.5}\}/2$, where ΔAbs is the absorbance of the complex at certain wavelength (obtained by subtraction of the absorption of the components from the spectra of the mixtures), C_{com} is the concentration of the complex, and $C^o{}_D$ and $C^o{}_A$ are initial concentrations of CHX_3 and amine, ε and *l* are extinction coefficient of the complex and the length of the cell which was used in the UV-Vis measurements, and K_{eff} is the effective formation constant of the complex.

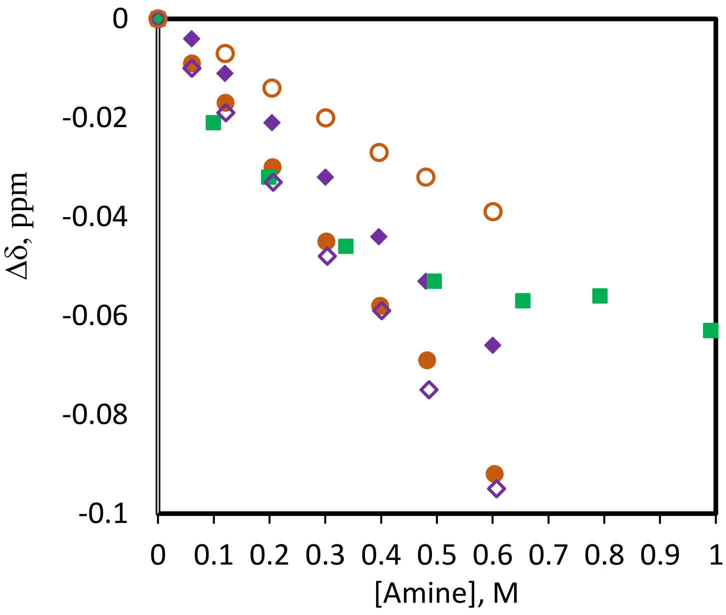

Figure S7. Dependencies of the chemical shifts of the protons of CHI_3 (as compared to that in the corresponding isolated molecules) on the concentration of added trimethylamine (TEA) (■), DMA (●), DMACN (○), DMABr (♦) or mMeODMA (◊) (in CD_3CN, 22°C).

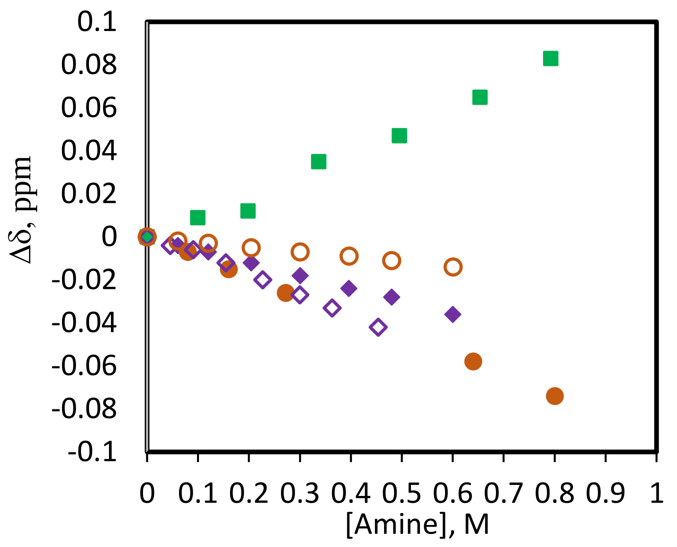

Figure S8. Dependencies of the chemical shifts of the protons of $CHBr_3$ as compared to that in the corresponding isolated molecules) on the concentration of added TEA (■), DMA (●), DMACN (○), DMABr (♦) or mMeODMA (◊) (in CD_3CN, 22°C).

.

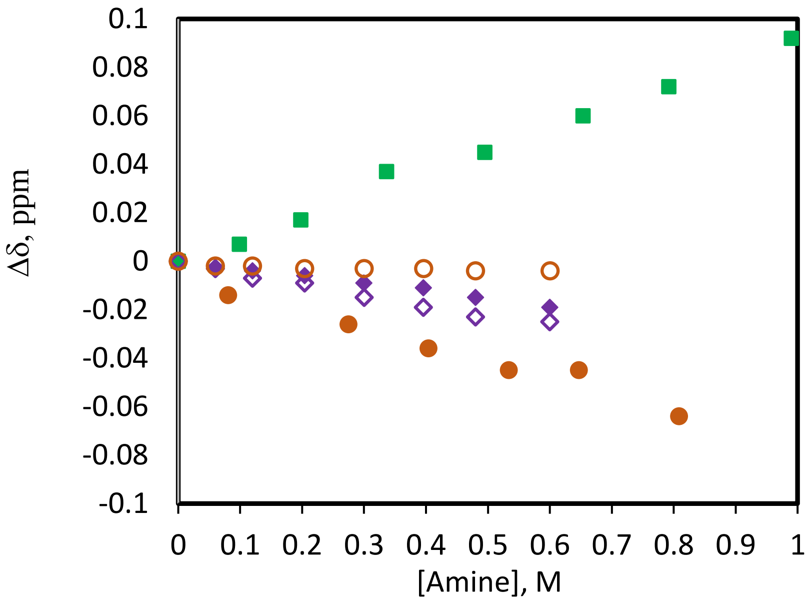

Figure S9. Dependencies of the chemical shifts of the protons of $CHCl_3$ as compared to that in the corresponding isolated molecules) on the concentrationof added TEA (■), DMA (●), DMACN (○), DMABr (♦) or mMeODMA (◊) (in CD_3CN, 22°C).

.

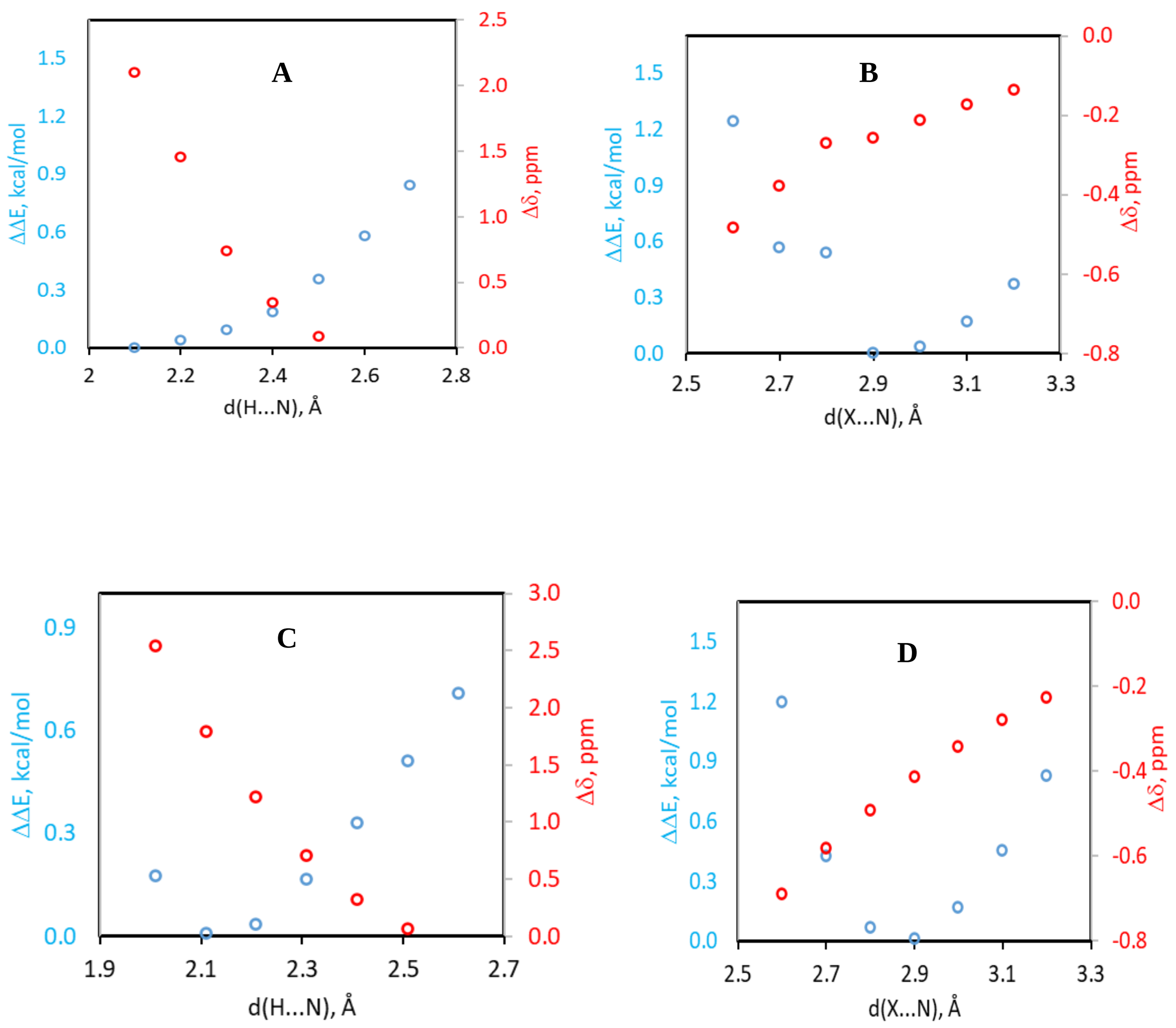

Figure S10. Effect of variations of interatomic H…N and X…N separations on the energies of HyB and HaB complexes (blue circle) relative to that of the energy minimum and the shift of the proton signal in their NMR spectra (red circles) relative to that in the individual CHX_3 for: A) HyB complexes of $CHCl_3$ with DABCO, B) HaB complexes of $CHCl_3$ with DABCO, C) HyB complexes of $CHBr_3$ with TMPD, D) HaB complexes of $CHBr_3$ with TMPD.

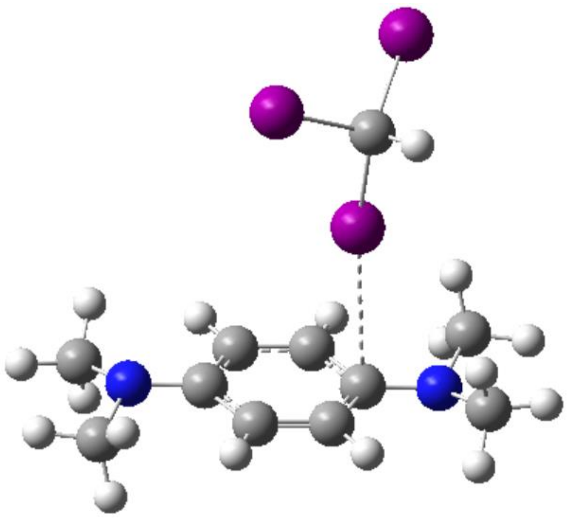

Figure S11. Alternative structure of the HaB complex between CHI_3 and TMPD showing I…C bonding ($d_{I...C}$= 3.35 Å, ΔE = -4.3 kcal/mol, chemical shift relative to that of the individual CHI_3 ΔΔδ = -0.55 ppm and absorption band with λ_{max} = 392 nm and ε = 2900 M^{-1} cm^{-1}).

Table S1. Values of V_{max} on the surfaces of halogen (V^X) and hydrogen (V^H) atoms (at 0.001 a.u. electron density) in the individual haloforms and in the molecules polarized by the presence of electron rich center nearby halogen (in HaB complex) or hydrogen (in HyB complex) atoms.

Molecule	V_{max}^H, a.u.	V_{max}^X, a.u.
CHI_3 (Ind)	0.0541	0.0425
CHI_3 (HaB complex)	0.0434	0.124
CHI_3 (HyB complex)	0.108	0.0334
$CHBr_3$ (Ind)	0.0611	0.0302
$CHBr_3$ (HaB complex)	0.0528	0.0828
$CHBr_3$ (HyB complex)	0.108	0.0181
$CHCl_3$ (Ind)	0.0624	0.02
$CHCl_3$ (HaB complex)	0.055	0.055
$CHCl_3$ (HyB complex)	0.103	0.0141

Table S2. Crystallographic, data collection and refinement details.

	CHI_3·DABCO	2CHI_3·TMPD	CHI_3·TMPD
Chemical formula	$C_6H_{12}N_2$·CHI_3	$C_{10}H_{16}N_2$·2(CHI_3)	$C_{13}H_{20}I_3N_3$
M_r	505.89	951.68	599.02
Crystal system, space group	Orthorhombic, *Pnma*	Triclinic, $P\bar{1}$	Orthorhombic, *Pnma*
Temperature (K)	150	150	150
a, *b*, *c* (Å)	16.4635 (13), 10.6079 (9), 7.3574 (6)	6.6114 (3), 9.6487 (5), 9.8861 (5)	10.5562 (5), 15.1118 (6), 11.6277 (5)
α, β, γ (°)		63.578 (2), 83.094 (3), 74.633 (3)	1854.89 (14)
V (Å^3)	1284.92 (18)	544.58 (5)	4
Z	4	1	Mo *K*α
Radiation type	Mo *K*α	Mo *K*α	5.05
μ (mm^{-1})	7.26	8.55	0.32 × 0.25 × 0.16
Crystal size (mm)	0.25 × 0.23 × 0.18	0.21 × 0.20 × 0.13	$C_{13}H_{20}I_3N_3$
Diffractometer	Bruker AXS D8 Quest CMOS diffractometer	Bruker AXS D8 Quest diffractometer with PhotonII charge-integrating pixel array detector (CPAD)	Bruker AXS D8 Quest diffractometer with PhotonII charge-integrating pixel array detector (CPAD)
Absorption correction	Multi-scan *SADABS* 2016/2: Krause, L., Herbst-Irmer, R., Sheldrick G.M. & Stalke D., J. Appl. Cryst. 48 (2015) 3-10	Multi-scan *SADABS* 2016/2: Krause, L., Herbst-Irmer, R., Sheldrick G.M. & Stalke D., J. Appl. Cryst. 48 (2015) 3-10	Multi-scan *SADABS* 2016/2: Krause, L., Herbst-Irmer, R., Sheldrick G.M. & Stalke D., J. Appl. Cryst. 48 (2015) 3-10
T_{min}, T_{max}	0.432, 0.747	0.500, 0.747	0.539, 0.747
No. of measured, independent and observed [$I > 2\sigma(I)$] reflections	13583, 3209, 2702	13429, 4038, 3603	52270, 3646, 3302
R_{int}	0.040	0.037	0.043
(sin θ/λ)$_{max}$ (Å^{-1})	0.833	0.771	0.769
$R[F^2 > 2\sigma(F^2)]$, $wR(F^2)$, S	0.037, 0.082, 1.06	0.022, 0.046, 1.05	0.013, 0.028, 1.06
No. of reflections	3209	4038	3646
No. of parameters	65	94	98
H-atom treatment	H-atom parameters constrained	H-atom parameters constrained	H-atom parameters constrained
$\Delta\rangle_{max}$, $\Delta\rangle_{min}$ (e Å^{-3})	2.58, -2.25	1.79, -1.73	0.50, -0.43

Computer programs: Apex3 v2016.9-0 (Bruker, 2016), *SAINT* V8.37A (Bruker, 2016), *SHELXS97* (Sheldrick, 2008), *SHELXL2017*/1 (Sheldrick, 2015, 2016), SHELXLE Rev714 (Hübschle *et al.*, 2011).

Table S3. Energies of the HyB and HaB complexes between CHX_3 and amines resulting from the M06-2X/def-TZVPP computations (acetonitrile).

CHX_3	Amine	Energy + ZPE, Hartree		BSSE, Hartree		ΔE, kcal/mol[a]	
		HaB complex	HyB complex	HaB complex	HyB complex	HaB	HyB
CHI_3	DABCO	-1276.702449	-1276.698772	0.000676	0.000626	-7.0	-4.7
	TMPD	-1431.471322	-1431.471276	0.000619	0.000635	-5.5	-5.5
$CHBr_3$	DABCO	-8106.386286	-8106.387885	0.000667	0.000793	-3.5	-4.4
	TMPD	-8261.157135	-8261.159697	0.000649	0.000902	-3.3	-4.7
$CHCl_3$	DABCO	-1764.400973	-1764.404661	0.000553	0.000832	-1.8	-4.0
	TMPD	-1919.172264	-1919.176305	0.000583	0.000940	-1.9	-4.2

a) $\Delta E = E_{comp} - (E_{CHX3} + E_D) + BSSE$ where E_{comp}, E_{CHX3} and E_A are sums of the electronic and ZPE of the complex, CHX_3 and amine and BSSE is a basis set superposition error. Energies of the individual molecules

Amines	Energy+ZPE, Hatree	CHX_3	Energy+ZPE, Hatree
TMPD	-499.89700337	CHI_3	-931.564856
DABCO	-345.125783	$CHBr_3$	-7761.254232
		$CHCl_3$	-1419.271718

Table S4. Atomic coordinates of the optimized HyB and HaB complexes between CHX_3 and amines (from the M06-2X/def-TZVPP computations in acetonitrile)

CHI_3-TMPD (HyB)				CHI_3-TMPD(HaB)			
C	-2.50128400	2.11661400	0.00105700	C	4.03883200	-1.62919400	-0.19174400
C	-1.57644800	1.66757400	0.93943400	C	3.08456700	-1.09818500	0.67069100
C	-1.84988200	0.44981200	1.56934800	C	3.22299400	0.24679500	1.02451500
C	-2.98718700	-0.28073700	1.28251700	C	4.27124500	1.01682000	0.55981500
C	-3.92871500	0.17240400	0.34032300	C	5.25125200	0.48036800	-0.29605500
C	-3.64249300	1.38379000	-0.30202000	C	5.09028700	-0.85828700	-0.67424200
H	-2.33789800	3.04428000	-0.52837300	H	3.97917900	-2.65897200	-0.51215900
H	-1.14420900	0.04616100	2.28556800	H	2.48246800	0.71350100	1.66084500
H	-3.13230400	-1.22035500	1.79379500	H	4.31687700	2.05175200	0.86281100
H	-4.31236000	1.77723500	-1.05140100	H	5.79099000	-1.32516000	-1.34950300
N	-5.08760500	-0.54463100	0.07146100	N	6.32508800	1.24121100	-0.73356300
N	-0.35376200	2.33474300	1.20942500	N	1.96788000	-1.84137200	1.14817600
C	-5.12405000	-1.93910000	0.46701900	C	6.23505500	2.68233100	-0.60525900
H	-6.07235200	-2.36821000	0.15593600	H	7.13594900	3.12864900	-1.01659600
H	-4.31083000	-2.52405900	0.01907800	H	5.36694700	3.09795600	-1.13153100
H	-5.05743100	-2.03788800	1.55001500	H	6.17043200	2.97594700	0.44216400
C	-5.83127600	-0.19567400	-1.12220800	C	7.10536200	0.73429900	-1.84404600
H	-6.69302600	-0.85196500	-1.20493100	H	7.89146300	1.44694600	-2.07677800
H	-6.20004900	0.82791300	-1.06264700	H	7.58196000	-0.21031900	-1.58280100
H	-5.22940600	-0.28969500	-2.03518200	H	6.49974000	0.57386700	-2.74481900
C	-0.12423600	2.66694500	2.61081600	C	1.81076300	-1.83606200	2.60140800
H	0.91328100	2.97331000	2.73885500	H	0.85120600	-2.28568000	2.85384100
H	-0.77488100	3.48689500	2.94285700	H	2.60881300	-2.40746400	3.09043400
H	-0.30513500	1.80404700	3.24553000	H	1.82115700	-0.82053000	2.98603300

C	-0.03044500	3.44703500	0.33726400	C	1.83287300	-3.18625400	0.61413700
H	0.98395200	3.77629800	0.55671800	H	0.89298900	-3.60580000	0.96921300
H	-0.07136600	3.12860500	-0.70432600	H	1.80648300	-3.15617000	-0.47398400
H	-0.70612600	4.30326900	0.47122800	H	2.64946000	-3.84617800	0.93265000
C	1.20460200	-0.17745600	0.00075000	C	-2.13490500	0.38666700	-0.61688200
H	0.64941700	0.50756900	0.63144500	H	-1.89526400	0.77196100	-1.59787300
I	1.33638000	-2.03381500	1.03579800	I	-0.36771600	-0.54943300	0.16219700
I	3.10101600	0.75706700	-0.27202300	I	-3.73270600	-0.99934600	-0.91601500
I	0.09541100	-0.32290200	-1.81124900	I	-2.74583400	2.07936500	0.53366200
$CHBr_3$-TMPD (HyB)				$CHBr_3$-TMPD (HaB)			
C	-1.98135200	1.92281000	-0.57393700	C	-3.31674500	1.63856500	-0.30699700
C	-1.09686600	1.72540100	0.48213500	C	-2.32496900	1.16970200	0.55126700
C	-1.39242300	0.69308200	1.37631400	C	-2.47435100	-0.13210100	1.04071100
C	-2.51342400	-0.10091900	1.22824300	C	-3.56359300	-0.91465800	0.70967500
C	-3.41394300	0.09738000	0.16553300	C	-4.57820700	-0.43809400	-0.14054200
C	-3.10571000	1.12275100	-0.73775700	C	-4.40936700	0.85225300	-0.65563300
H	-1.79854800	2.69787900	-1.30434300	H	-3.25350000	2.63142200	-0.72812000
H	-0.71357500	0.48438500	2.19429000	H	-1.70856400	-0.55598900	1.67692500
H	-2.67668900	-0.89186400	1.94447900	H	-3.61387200	-1.91422100	1.11416800
H	-3.74402800	1.31419300	-1.58686300	H	-5.13673400	1.27059800	-1.33465700
N	-4.55525100	-0.68210100	0.02837100	N	-5.69440700	-1.20960000	-0.44247200
N	0.11241200	2.45485400	0.63603000	N	-1.17374300	1.92272400	0.88742900
C	-4.60345100	-1.93951600	0.74893100	C	-5.61521700	-2.63627300	-0.19723100
H	-5.53449600	-2.44581700	0.51023700	H	-6.54688500	-3.09883900	-0.51084700
H	-3.76789400	-2.60284600	0.49228900	H	-4.78833500	-3.11181000	-0.73953200
H	-4.58638300	-1.77184000	1.82533800	H	-5.48757900	-2.84138500	0.86518500
C	-5.24445100	-0.64540900	-1.24585100	C	-6.51261600	-0.79552600	-1.56431000
H	-6.09704900	-1.31738500	-1.20465600	H	-7.32724500	-1.50385000	-1.68654400
H	-5.62222700	0.35546500	-1.45265500	H	-6.95181000	0.18486000	-1.38162700
H	-4.59966900	-0.94705200	-2.08117900	H	-5.94641500	-0.74654700	-2.50324400
C	0.25485900	3.13996500	1.91643200	C	-0.89514700	2.04019800	2.31307500
H	1.27936700	3.49498400	2.01966400	H	0.10391300	2.45450400	2.44528700
H	-0.42296300	4.00084000	1.98903600	H	-1.61697400	2.69889100	2.81333400
H	0.04335000	2.46324500	2.73941200	H	-0.92220800	1.06686100	2.79412300
C	0.47246200	3.32038900	-0.47107800	C	-1.01660400	3.19188400	0.20594200
H	1.47268600	3.71236000	-0.29433500	H	-0.03756000	3.59907900	0.45439300
H	0.48260900	2.75222500	-1.40101700	H	-1.06407800	3.04745900	-0.87245800
H	-0.21326000	4.17108200	-0.58607700	H	-1.77900200	3.92740100	0.49673200
C	1.59416800	-0.31693800	-0.01764700	C	2.63916100	-0.60291600	-0.58444300
H	1.15017200	0.57930300	0.40462700	H	2.35454800	-1.18304800	-1.44966200
Br	1.58185300	-1.69123200	1.33638400	Br	3.22268200	-1.85380000	0.76837100
Br	0.52726300	-0.81774300	-1.54374200	Br	1.09766900	0.40751800	-0.00231000
Br	3.39457700	0.14976400	-0.53798300	Br	4.08551300	0.55612700	-1.13169600
$CHCl_3$-TMPD (HyB)				$CHCl_3$-TMPD (HaB)			
C	-1.08545100	1.70398500	-0.79175900	C	-2.15301000	1.63525800	-0.54977700
C	-0.21799500	1.50518600	0.27829300	C	-1.11957400	1.26555200	0.30987800

C	-0.61950500	0.59976600	1.26397100	C	-1.28189000	0.06365500	1.00908700
C	-1.82722100	-0.06792600	1.19265000	C	-2.41677000	-0.71270200	0.87225300
C	-2.71259900	0.13680000	0.11849000	C	-3.46913600	-0.33333100	0.02009800
C	-2.29707600	1.02886000	-0.87855700	C	-3.29158000	0.85160100	-0.70236100
H	-0.82252800	2.38081700	-1.59204300	H	-2.08659900	2.54653400	-1.12602900
H	0.04098500	0.38906600	2.09645100	H	-0.49192100	-0.29175900	1.65692400
H	-2.07246800	-0.76483900	1.97963500	H	-2.47318700	-1.63270500	1.43418300
H	-2.91885600	1.21315200	-1.74141500	H	-4.04812200	1.19051000	-1.39376100
N	-3.94050300	-0.50873600	0.05845500	N	-4.63036100	-1.09422900	-0.08590000
N	1.06674300	2.10451200	0.36606100	N	0.07191300	2.01039500	0.44255900
C	-4.13484700	-1.67149600	0.90227100	C	-4.57303700	-2.47129300	0.36442700
H	-5.12484900	-2.07862600	0.71711800	H	-5.53788900	-2.93890000	0.18841700
H	-3.39213000	-2.45674300	0.71363800	H	-3.80165400	-3.05327100	-0.15587300
H	-4.08235300	-1.39983900	1.95615900	H	-4.37353500	-2.52224200	1.43420500
C	-4.63935800	-0.50911200	-1.21092600	C	-5.49923700	-0.83081000	-1.21517700
H	-5.56505600	-1.06743400	-1.10339600	H	-6.34583100	-1.51064800	-1.17444300
H	-4.89861900	0.50607900	-1.51021600	H	-5.88990500	0.18546400	-1.17375900
H	-4.04768300	-0.96240800	-2.01639400	H	-4.98989800	-0.96201700	-2.17872000
C	1.29193000	2.88939200	1.57539100	C	0.50658400	2.28605000	1.80372300
H	2.34984100	3.13780900	1.64851100	H	1.52302200	2.67740200	1.77764300
H	0.71354000	3.82280500	1.56198100	H	-0.13981300	3.02467300	2.29646900
H	1.01218300	2.32244200	2.45874700	H	0.51386300	1.37964400	2.40179600
C	1.51324500	2.81592000	-0.81660900	C	0.21008700	3.16777100	-0.41566500
H	2.55323400	3.10579700	-0.67579400	H	1.21722800	3.56556400	-0.30116700
H	1.44979000	2.16658800	-1.68932500	H	0.07183000	2.88312900	-1.45778700
H	0.92831100	3.72531100	-1.01195900	H	-0.50460100	3.96701300	-0.17356100
C	2.16194200	-0.89248500	-0.07565700	C	3.47654500	-0.97417700	-0.63478600
H	1.88788300	0.08208100	0.31756000	H	3.24265800	-1.46755300	-1.56772000
Cl	3.86229400	-0.82264900	-0.54963600	Cl	2.18866200	0.17183100	-0.26614300
Cl	1.91136300	-2.11030500	1.17661700	Cl	3.59362000	-2.21257200	0.61934600
Cl	1.13850300	-1.21562400	-1.47558200	Cl	5.02298900	-0.14393400	-0.83282700
$CHBr_3$-DABCO (HyB)				$CHBr_3$-DABCO (HaB)			
C	-1.22279200	0.00045700	0.08015600	C	2.18262800	-0.00010400	-0.56732600
N	1.85197500	0.08602100	0.40700900	N	-2.52276100	-0.01376500	-0.02562200
C	2.16656300	0.60389700	-0.93149800	C	-3.08030000	1.03518400	-0.88396400
C	2.59312000	0.86822800	1.40397700	C	-3.06829800	-1.31072500	-0.43556200
C	2.28103500	-1.31689800	0.48571800	C	-2.90274200	0.25035000	1.36489500
N	4.36400500	-0.09053400	-0.03407200	N	-5.06516800	0.01369500	0.18441200
H	1.52899200	0.09715100	-1.65833700	H	-2.60108700	1.98093500	-0.62736100
H	1.91307700	1.66475500	-0.94720500	H	-2.82119600	0.79697800	-1.91659800
H	2.19489000	1.88330500	1.41418700	H	-2.70598400	-1.53460900	-1.43980000
H	2.40865900	0.42466000	2.38305200	H	-2.67370100	-2.07022400	0.24079500
H	1.94565000	-1.72928800	1.43807900	H	-2.41065400	-0.48542100	2.00227600
H	1.77757900	-1.86450500	-0.31264000	H	-2.52276800	1.23648400	1.63545000
C	3.67112900	0.37650800	-1.23961500	C	-4.62014500	1.10589300	-0.68733100
H	4.14530700	1.29723300	-1.58046500	H	-5.14237500	1.02310000	-1.64092200
H	3.80344200	-0.37704400	-2.01692200	H	-4.91388900	2.04859200	-0.22433500

C	4.10646900	0.85692400	1.05565000	C	-4.62082800	-1.26097000	-0.38887100
H	4.70584300	0.56689800	1.91905100	H	-5.02183400	-2.07287500	0.21849200
H	4.44158400	1.84248300	0.73024400	H	-5.04659600	-1.34794900	-1.38911600
C	3.82423800	-1.40108500	0.34260500	C	-4.44891000	0.18017200	1.50468800
H	4.10880600	-2.12816800	-0.41846600	H	-4.84414200	1.08891300	1.95950100
H	4.29024000	-1.69936000	1.28249400	H	-4.74886400	-0.66428200	2.12619200
H	-0.18913200	0.00662700	0.42780500	Br	2.99463900	1.60008400	0.15434800
Br	-1.33098000	-1.32002100	-1.32633200	Br	3.01291500	-1.58934800	0.15759300
Br	-1.58508300	1.77391800	-0.59059300	Br	0.26495700	-0.01067700	-0.30813100
Br	-2.37266100	-0.45050700	1.56111400	H	2.38227300	-0.00001100	-1.62861600
$CHCl_3$-DABCO (HyB)				$CHCl_3$-DABCO (HaB)			
C	2.03809100	0.00013900	0.10152100	C	3.08696500	-0.00121800	-0.50344800
N	-1.05816600	0.05327600	0.36557000	N	-1.58488400	-0.29863800	-0.39288100
C	-1.52148100	-1.34038800	0.38763200	C	-1.82258800	1.14122000	-0.53013200
C	-1.82733200	0.82698200	1.34765300	C	-2.61855000	-1.02498900	-1.13660400
C	-1.29961900	0.61114800	-0.97164600	C	-1.67592500	-0.66055600	1.02491000
N	-3.55155600	-0.05294700	-0.19332700	N	-3.90046400	0.29819900	0.51715900
H	-0.99370600	-1.87967000	-0.40043800	H	-1.00315200	1.67322400	-0.04420000
H	-1.24121800	-1.78461200	1.34337300	H	-1.79715700	1.38672700	-1.59284800
H	-1.69672700	0.35564100	2.32243300	H	-2.49234400	-0.81537000	-2.19934800
H	-1.40865300	1.83244700	1.40169100	H	-2.45648500	-2.09268600	-0.98331200
H	-1.02062800	1.66540600	-0.94944200	H	-1.41365600	-1.71422800	1.12984600
H	-0.64222000	0.10666100	-1.68231500	H	-0.93235500	-0.07554800	1.56858000
C	-3.05809700	-1.38520900	0.17091400	C	-3.19199000	1.51359300	0.10211300
H	-3.57411800	-1.69812500	1.07944300	H	-3.81928400	2.05474300	-0.60707900
H	-3.32264800	-2.08450000	-0.62276800	H	-3.06049100	2.14410700	0.98243000
C	-3.32346000	0.85865400	0.93287500	C	-4.02690100	-0.59320200	-0.64063200
H	-3.62330400	1.85991600	0.62115500	H	-4.62360100	-1.45798500	-0.34837300
H	-3.96688100	0.55827300	1.76035600	H	-4.57111200	-0.05813800	-1.41995800
C	-2.79378600	0.42985100	-1.35273800	C	-3.11046200	-0.37919500	1.55078200
H	-2.90897300	-0.29809100	-2.15674200	H	-3.08759300	0.25329200	2.43895300
H	-3.23016300	1.37135000	-1.68750800	H	-3.62223500	-1.30619800	1.81264900
H	1.03095200	0.00459200	0.51621500	H	3.46990900	-0.00160700	-1.51431100
Cl	2.29464100	1.56403300	-0.67893500	Cl	1.33974300	-0.21832700	-0.56115200
Cl	2.11062500	-1.30552500	-1.08766700	Cl	3.85998300	-1.32897000	0.36894300
Cl	3.21711300	-0.25960500	1.38728300	Cl	3.50402100	1.54958000	0.23368500
CHI_3-DABCO (HyB)				CHI_3-DABCO (HaB)			
C	-0.79508700	-0.03847900	-0.02185900	C	1.72078000	-0.00017000	-0.61980300
N	2.26804100	0.09817400	0.50236500	N	-3.09669500	-0.00628500	-0.02812100
C	2.72131600	0.35538500	-0.87118600	C	-3.66314700	1.23231400	-0.57776400
C	2.88736600	1.08290300	1.39768600	C	-3.69298700	-1.15873900	-0.71629100
C	2.70130500	-1.24782000	0.90023300	C	-3.40531300	-0.08570800	1.40570800
N	4.81497200	-0.07987200	0.37032200	N	-5.62176000	0.00611700	0.30863500
H	2.16827300	-0.29968600	-1.54749200	H	-3.14412500	2.07710000	-0.12384800
H	2.46375400	1.38563500	-1.12029000	H	-3.45787300	1.24646300	-1.64871000
H	2.47654000	2.06676600	1.16833500	H	-3.37822100	-1.13467200	-1.75999500

H	2.60480600	0.83045100	2.42041500	H	-3.28820400	-2.06411300	-0.26273300
H	2.26586100	-1.47968400	1.87303000	H	-2.90097200	-0.96097800	1.81644800
H	2.29689400	-1.95556800	0.17475500	H	-2.98819900	0.80002600	1.88623100
C	4.25173400	0.11559200	-0.96995100	C	-5.18827600	1.27321000	-0.28689300
H	4.75167400	0.96337800	-1.43921300	H	-5.75521000	1.43955000	-1.20276100
H	4.47336100	-0.77383900	-1.56102100	H	-5.43109600	2.07568800	0.40989200
C	4.42932800	1.05569700	1.21528000	C	-5.23927400	-1.09323900	-0.58211800
H	4.93692700	0.96415600	2.17593000	H	-5.63472100	-2.02410700	-0.17590100
H	4.78368300	1.96824000	0.73434800	H	-5.70812400	-0.92282600	-1.55145700
C	4.25180800	-1.30680200	0.94358600	C	-4.94455900	-0.16923300	1.59654200
H	4.62815100	-2.16082500	0.37976700	H	-5.29447500	0.60246700	2.28204700
H	4.61322500	-1.39599500	1.96875500	H	-5.23534900	-1.13791600	2.00336700
H	0.22370400	0.00004400	0.36515800	H	1.92864200	0.00009000	-1.68096000
I	-0.89900500	-1.81078800	-1.20219900	I	-0.42196800	-0.00685600	-0.34561400
I	-1.00768800	1.73364200	-1.18744300	I	2.64937600	-1.76858300	0.14289000
I	-2.11086600	-0.08420700	1.65216700	I	2.63662500	1.77569900	0.14099800
